# Navigating Homelessness Assistance While Pregnant: A Rapid Qualitative Research-to-Policy Collaboration in Washington, DC

**DOI:** 10.1089/heq.2023.0235

**Published:** 2024-05-30

**Authors:** Christina X. Marea, C. Anneta Arno, Kelly Sweeney McShane, Andrew Lozano, Makeda Vanderpuije, Kelley N. Robinson, Karen Trister Grace, Noelene Jeffers

**Affiliations:** ^1^ School of Nursing, Georgetown University, Washington, District of Columbia, USA.; ^2^ Office of Health Equity, DC Department of Health, Washington, District of Columbia, USA.; ^3^ Community of Hope, Washington, District of Columbia, USA.; ^4^ School of Nursing, University of Maryland , Baltimore, Maryland, USA.; ^5^ School of Nursing, George Mason University, Fairfax, Virginia, USA.; ^6^ School of Nursing, Johns Hopkins University, Baltimore, Maryland, USA.

**Keywords:** homelessness, housing insecurity, pregnancy, perinatal, health equity, health disparities

## Abstract

**Background:**

Homelessness during pregnancy contributes to adverse pregnancy and infant outcomes from birth through early childhood. Washington, DC, a microcosm of structural inequities in the United States, has persistent racial disparities in perinatal outcomes and housing insecurity.

**Methods:**

Grounded in a reproductive justice framework, we explored the lived experience of navigating homelessness assistance while pregnant to inform recommendations for a collaborative policy and practice change effort. We conducted 20 individual interviews with DC residents who experienced homelessness during pregnancy. We analyzed the data using thematic analysis and an action-oriented approach.

**Results:**

Our analysis resulted in three main recommendation areas for policy and practice change: (1) timely and meaningful access to safe and stable housing in pregnancy; (2) care coordination for services and referrals that support physical, mental, and social well-being; and (3) access to a living wage and affordable housing.

**Discussion:**

Access to stable housing is critical to ensure that pregnant and parenting people can have and raise children in a safe and sustainable environment—key tenets of reproductive justice. Housing support must be meaningfully accessible, including service delivery that accommodates the complex social histories and competing demands that accompany housing insecurity.

**Health Equity Implications:**

This study informed the development of strategic recommendations, catalyzed a new model for multisector collaboration, and influenced a system-wide practice change to expand access to robust housing supports for pregnant people. Policy and practice change require sustained leveraging of political will to promote economic justice and ensure that residents can achieve safe, sustainable, and affordable housing.

## Introduction

In 2022, more than half a million people in the United States were experiencing homelessness, which includes those in temporary or transitional shelters and unsheltered people staying outdoors or other places not meant for human habitation.^1^ Between 2016 and 2020, trends in unhoused status (lack of fixed regular, adequate nighttime residence) during pregnancy worsened with rates increasing by 70% nationally during that period.^2^ These estimates do not always account for all forms of housing insecurity (HI), which include housing problems people may experience including affordability, safety, quality, insecurity, and permanent loss of housing (homelessness). The lack of affordable housing, race- and gender-driven pay gaps, and historical U.S. housing policies are structural factors linked to increased HI, especially among metropolitan communities, with increases even higher among Black people.^3^ HI contributes to adverse maternal, perinatal, and infant and child health outcomes driven by decreased access to and utilization of health care, chronic stress, and environmental exposures.^4,5^

Research focusing on women experiencing HI has found higher rates of depression, stress, post-traumatic stress disorder, and comorbid mental disorders compared to those with stable housing—issues that persist in pregnancy.^6,7^ Gaps in health care due to HI can lead to increased pregnancy-related emergency department (ED) visits, birth complications, hospital readmissions, chronic health conditions, higher health care costs, and maternal morbidity.^2,8,9^ HI is also associated with poor infant outcomes including preterm birth and low birth weight, neonatal intensive care unit admissions, ED visits, and worse acute and chronic health conditions through at least 6 years of age.^10,11^

Black birthing people bear a disproportionate burden of HI and adverse perinatal health outcomes due to structural racism—defined as macrolevel infrastructure and policies that reduce access to opportunities, resources, and power—including affordable housing.^12–14^ Racial residential segregation, the separation of people’s residences according to socially constructed race or ethnicity, is associated with lower-quality housing and increased exposure to environmental contaminants that contribute to adverse perinatal outcomes.^15,16^ In the United States, racial residential segregation has been driven by private and public socioeconomic policies that exclude people socially designated as “non-White.” Both HI and exposure to racism (i.e., Black/African descent as a proxy measure) are associated with higher rates of hypertensive disorders of pregnancy, postpartum hemorrhage, preterm birth, and low birth weight.^17,18^ Infants exposed to HI are less likely to receive well-child care and are at increased risk for acute and chronic health challenges for at least 6 years beyond the period of homelessness.^11,19^ Estimates of homelessness prevalence in pregnancy and at the time of delivery range from 0.3% to 4%, with Black birthing people experiencing twice the rate of HI compared with their White counterparts.^20,21^ Washington, DC, where 86% of people experiencing homelessness are Black, is a microcosm of complex intersections between homelessness, perinatal health, and racial inequities.^22^ This research adds to our knowledge about the growing challenges faced by pregnant people experiencing HI in metropolitan areas and elevates their voices to inform policies and practices to improve housing, social, and medical support.

The purpose of this study was to understand pregnant people’s experiences navigating homelessness assistance and to inform and rapidly generate public policy and practice change recommendations to address the needs of this structurally marginalized group. This study was embedded within the DC Calling All Sectors Initiative (CASI), a philanthropically funded initiative led by the DC Health Office of Health Equity (OHE). CASI was a multisector collaboration that aimed to address structural inequities and barriers at the intersection of pregnancy and homelessness. DC Health OHE partnered with Community of Hope (a federally qualified health center and homelessness service provider in DC) and key government agencies to generate innovative policies and practices to catalyze system-level change to address HI and poor perinatal outcomes.

## Methods

### Study design

We used an action-oriented qualitative research approach, designed to rapidly inform policy recommendations.^23^ This study was approved by the Georgetown University Medical Center institutional review board (Study #00004530).

### Conceptual framework

This study was grounded in the reproductive justice framework that affirms the human right to (1) have children, (2) not have children, and (3) parent children in safe and sustainable communities.^24^ Inequities in housing, particularly along racial lines, are caused by policies and practices grounded in mutually reinforcing systems driven by structural racism and represent an affront to the principles of reproductive justice.^25^ This framework is critical to the study design and analysis because it requires consideration of how experiencing homelessness affects the participants’ ability to achieve reproductive justice, and how structural systems uplift or inhibit participants’ access to these human rights at a time when they are highly vulnerable. This framework informed the development of the interview guide as we sought to understand the factors that led to experiencing homelessness while pregnant, including the role and timing of the pregnancy relative to becoming unhoused (right to have or not have children) and how people were able (or not able) to access housing and other social assistance to support their well-being (right to parent in safe and stable communities). This framework further grounded our analysis as we became deeply immersed in the client voices—what they said they needed to feel safe and well during pregnancy and as parents.

### Participants and recruitment

Participants were eligible to participate if they were 18 years of age or older, had experienced homelessness during or within 3 months of the end of a pregnancy in the last 5 years, spoke fluent English, and had access to a phone/smartphone or computer. We used a purposive sampling strategy to enroll individuals who met inclusion criteria and were likely to have insights into the experiences of navigating homelessness assistance in DC. The CASI team sent study information to DC homelessness services agencies, who shared the information with eligible clients (see Supplementary Appendix S1—Agencies Contacted). Interested participants conducted eligibility screening through the enrollment website or by phone, then scheduled an interview, and were emailed information about the study. Participants received a $75 retail gift card to thank them for their time and expertise.

### Data collection

We developed the qualitative interview guide (see Supplementary Appendix S2–Interview Guide) based on the aims of the study and the theoretical framework informing our approach. Questions explored the experience of homelessness during pregnancy, services that were helpful or inhibitory, experiences seeking help with housing, strategies used to meet basic needs, and recommendations for improving support services. Two authors, unknown to participants, with training and experience in qualitative research and the clinical care of marginalized groups conducted the interviews. They are PhD-prepared certified nurse-midwives, identify as women, and gave birth at least once since 2018. They conducted the interviews by phone or secure video conferencing platform (Zoom) at a time convenient to the participants. They obtained oral informed consent and audio-recorded each interview, which lasted 40–90 min. Interviews were professionally transcribed and error-checked.

### Analysis

During the data collection period, the two interviewers met weekly to review content and themes, noting new areas that emerged. At interview 16, the interviewers determined that saturation had been achieved and then conducted four more interviews to confirm that no new themes would arise. We used directed content analysis with an action-oriented approach to guide data analysis, which was conducted on transcripts uploaded to the Dedoose qualitative analysis web application.^26,27^ Two authors who coded the data also conducted and read all interviews and were thus fully immersed in the data. One author created the preliminary codebook by inductive coding of one transcript. A second author then applied the codebook to the same transcript, adding codes as needed. Both authors then met to review, compare, and clarify the codes and identify main categories/subcategories with associated summaries and anchor codes. Each author then independently coded another two transcripts and met again to review the coding until they achieved >80% consensus on code application. The coding authors then applied the finalized codebook to all interview transcripts. We checked whether any theme existed within a single interview (none), confirming that data saturation had been achieved. Next, the coding authors used inductive abstraction to identify themes, which were grouped together to result in the three recommendation areas for policy and practice change. Key quotes from participants in each area were used to highlight the resident experiences that would be alleviated by the practice change recommendations presented in the tables.

## Results

### Participant characteristics

We conducted 20 in-depth individual interviews. All participants self-identified as Black/African American and as women. One-third of participants were currently pregnant and half had been pregnant within the last 2 years. See Table 1 for additional participant characteristics.

**Table 1.** Participant Demographic Characteristics (*n* = 20)

Characteristics	% (*N*)
Race	
Black/African American	100 (20)
Pregnancy	
Currently pregnant	35 (7)
Pregnant within the last 2 years	50 (10)
>2 years postpartum	15 (3)
Attempted to access DC homelessness services	
Yes	85 (17)
No	15 (3)
Employment status	
Part-time	25 (5)
Full-time or student	15 (3)
Unemployed	60 (12)
Current age, mean (range)	7.%2 (19–35)
Emerging adult (18–24)	55 (11)
Adult (25+)	45 (9)
Age at first unstable housing	4.2 (4–33)
Childhood (<18)	30 (6)
Emerging adult (18–24)	55 (11)
Adult (25+)	15 (3)

Source: Participant demographic questionnaire.

### Themes

We present the findings here according to the three broad policy recommendation areas that the research team presented to the DC CASI working group in May 2021. Within each broad policy recommendation area, we highlight practice change recommendations and key quotes from participants who informed these recommendations.

**Policy Recommendation #1:** Ensure meaningful access to homelessness assistance for people who are pregnant, including the elimination of barriers that impede timely access. See Table 2 for practice change recommendations and key quotes from participants.

**Table 2.** Policy Recommendation #1: Ensure Meaningful Access to Homelessness Assistance for People Who Are Pregnant, and Eliminate Barriers That Impede Timely Access

Practice change recommendations	Key quotes (*participant age, education, age at first HI, and reproductive phase*)
**Theme #1.1:** *HI and trying to figure things out*	
Ensure pregnant people have immediate access to homelessness assistance, including during first, second, and third trimesters of pregnancy	. . . My biggest issue is [*housing office*] does not offer any help until the mother is six months or more. That’s very frustrating because with medical needs and stuff like that, I can’t wait for 6 months. I need stuff now . . . I was so scared for myself. *34 years old, Associate’s degree, First HI 18 years old, Pregnant*
	It was very depressing . . . when I was experiencing homelessness, I always kept a good job, so in the housing program’s eyes, [I] don’t need help. I couldn’t get housed until I was nine months pregnant . . . [the housing office said] “We can’t give you anything unless you’re seven months at least.” *24 years old, High school, First HI 18 years old, Postpartum*
	If I were to get into the shelter sooner when I initially thought that I was pregnant, that would have took a little bit of stress off, if I was able to find a job a little bit sooner so I could start saving for a baby, or some type of financial support, that would have been better. If I was able to make my own meals or eat healthy*. 32 years old, Some college, First HI 30 years old, Postpartum*
**Theme #1.2:** *Complex requirements are a barrier to access*	
Amend homelessness verification process to ensure timely access to services, and eliminate barriers created by bureaucratic processes.	When I first went in [to homelessness assistance], I was pregnant, probably four months . . . they was like, “we have to prove that you’re homeless . . . we'll contact your mom, your dad, grandma, aunties, and uncles.” I was like, “Then contact them. They already said I can’t go back to the house.” . . . They just let me go because they said they can’t prove that I’m homeless. It just brought me down even more just so that I couldn’t do nothing for myself. . . . [You] have to catch the bus [with] all your belongings to just sit up there all day and possibly not even get help. *25 years old, Vocational training, First HI 20 years old, Postpartum*
	When you’re pregnant, you are going through all types of emotions, you cry . . . you just feel vulnerable and you feel lost . . . and the baby feels what you feel . . . You never know what mental state people are in. I feel they should be better at connections and resources instead of just brushing people off . . . I’ve been up [to homelessness assistance] like five, six times. I have nowhere to go. I sleep in my car. I need help. *26 years old, Some college, First HI 22 years old, Pregnant*
	They said we didn’t qualify because we were on a lease before. We did get off of [the lease], and they still was like, “Oh, well you can stay with your friend.” They didn’t want to help. They just kept saying, “Oh, if your friend say you can stay with them, we’ll pay their utilities. We’ll buy groceries.” They was trying to do everything, BUT help . . . It doesn't always work out like that. You can’t always stay with someone. *26 years old, High school, First HI 18 years old, Pregnant*
**Theme #1.3:** *Importance of client-centered and trauma-informed housing support*	
Ensure respectful and responsive communication from homelessness assistance agencies and staff.	For better support, you need to have a little more patience. That’s all. A little more patience and not say act like you care but actually care about the pregnant person that you’re encountering. Actually, care about the pregnant females that are out here and know that everybody's circumstances are different. *24 years old, High school diploma, First HI 16 years old, 2 years postpartum*
	I feel like you shouldn’t just act the person out because they’re young or you feel as though they’re irresponsible . . . There should always be someone there to listen to them and be there for them . . . You know what I’m saying? Everybody needs some type of person, some type of support . . . I feel as though . . . [the staff looks] at everyone as a liar or a little girl who was being too grown and run away from home and got pregnant. That’s not how the case could always be. People go through stuff . . . and now everyone’s against them. They have no one by their side. *24 years old, 9th grade, First HI 16 years old, Postpartum*

HI, housing insecurity.

#### Theme #1.1 Housing uncertainty and trying to figure things out

All participants reported immense feelings of stress and anxiety related to the uncertainty of obtaining safe and stable housing. Participants actively problem-solved ways to find shelter, including relying on short-term solutions like “couch surfing” (staying with friends or relatives) and moving between short-term shelters. Some participants slept outside, where they were exposed to extreme weather elements, violence, theft, and injury. Among those able to access shelters, many were afraid of other shelter residents because of fear of theft and physical violence.

Participants strongly believed that pregnant people experiencing homelessness should have access to family housing as early in the pregnancy as possible to establish a stable foundation, and were frustrated by policies restricting access to family housing until the third trimester. Housing stability was described as an important precursor to obtaining medical care and other social services. By the third trimester, participants felt they had been accumulating stress and perinatal risk and had not been caring for themselves well.

#### Theme #1.2 Complex requirements are a barrier to access

Most participants attempted to access homelessness assistance and other social services during pregnancy. Participants described profound levels of vulnerability and insecurity, which made navigating complex requirements feel insurmountable. Many described anticipatory anxiety before attempting to access services—worries that they would not be believed or would not have all the needed documentation and thus be denied help. The lack of integration between health, housing, and social services often felt frustrating and burdensome with participants reporting a sense of hopelessness that they would ask for help and be told they were in the wrong place for that service. The lack of clear timelines for communication left many feeling as though they were constantly waiting with no idea when they might receive assistance.

Many felt that there was always one more hurdle, and the helpfulness of the response varied by staff member. Being listed on an existing lease was a frequent reason noted for being denied services; however, women shared that they were forced out by a family member. Being denied help owing to a perceived technicality, while alienated from family support, led to feelings of despair. Unclear information about program eligibility requirements, uncertain timelines for housing availability/length of stay, and what would happen at the end of a shelter/housing program contributed to a pervasive sense of instability that made it difficult to plan and prioritize, navigate other social services, find work, or focus on pregnancy health. There often was no explicit communication plan, leaving people unsure of next steps, frequency of contact to expect, and the roles of the individual versus an agency. Participants described being treated with impatience, or dismissal when seeking services, which, for some, resulted in desperate outbursts. Some participants felt hopeless.

Participants expressed two central desires in accessing social services—1) to be treated as a whole person who is struggling and deserving of care and 2) clear instructions on how to access the care, services, or resources they need. Participants recommended enabling pregnant people to self-attest that they no longer reside at an address where they may be listed on someone else’s lease (i.e., a pregnant person’s mother has kicked them out of the house but refuses to remove them from the lease). They emphasized the importance of trusting pregnant people when they state that they cannot live with a friend or family member; many have experienced abuse or neglect with their families of origin. These environments may be unsafe, unstable, and inappropriate for the pregnant person and/or a newborn. The participants deeply desired clear road maps for eligibility and communication, including explicit roles and timelines for status updates.

#### Theme #1.3 Importance of client-centered and trauma-informed housing support

Experiences with homelessness assistance services were characterized by inconsistent support and a perceived lack of compassion. Accessing homelessness assistance typically meant waiting in long lines, sometimes with children in tow, and frequently ending the day without the help they needed. Some participants reported that the COVID-19 era allowances for remote enrollment were helpful, but they feared that these changes would not be permanent. Women expressed a strong desire to be treated with care and respect, rather than as “cases” or stereotypes, by service providers. Women who received timely follow-up and anticipatory guidance about their case status appreciated it deeply. Unfortunately, more women reported experiencing disinterested or apathetic responses, late or no response to inquiries, infrequent communication, outdated information, and combative conversations. This treatment resulted in feelings of shame and anger.

Participants recommended that pregnant people should be eligible for permanent family housing as early in pregnancy as possible. They stated that the third trimester eligibility requirement was too late and that homelessness itself made their pregnancy high risk. The lack of housing in pregnancy presented logistical challenges such as no mailing address to receive insurance documents or obtain an identification—both required to access health care. Beyond logistical burdens, the extreme stress and fear of not having a safe place to live were persistent and intrusive with many participants attributing adverse pregnancy outcomes to their high levels of stress. Participants also desired communication characterized by a caring interpersonal demeanor, timely follow-up, and that they be believed and validated by agency staff.

**Policy Recommendation #2:** Ensure all pregnant people seeking homelessness assistance receive care coordination and referrals to support their physical, mental, and social well-being. See Table 3 for practice change recommendations and key quotes from participants.

**Table 3.** Policy Recommendation #2: Ensure All Pregnant People Seeking Homelessness Assistance Receive Referrals to Services to Support Their Physical, Mental, and Social Well-Being

Practice change recommendations	Key quotes (*participant age, education, age at first HI, and reproductive phase*]
**Theme #2.1:** *Meeting basic needs*	
Provide care coordination and/or case management to ensure pregnant people who are seeking homelessness assistance are able to meet their basic needs for health, hygiene, nutrition, communication, transportation, and safety.	[It’s] pretty scary because I'm not eating . . . My baby isn’t getting nutrients . . . I’m diabetic. That makes me a high-risk pregnancy . . . I really didn’t know [where] to go to get daily food or daily showers . . . It was difficult to keep my weight up and my mental state together . . . With diabetes, you’re supposed to have multiple small meals throughout the day . . . but I don’t have a refrigerator. *34 years old, Associate’s degree, First HI 18 years old, Currently pregnant*
	Every time they assigned me to different caseworkers. Some would want to really truly help but they require a lot of things to move forward . . . Then when I come back they need something else . . . all I've been doing is going back and forth on the train or the bus. *23 years old, Some college, First HI 18 years old, Postpartum*
	What I really needed was for someone to actually hear me, as a single parent having a young kid . . . I wish I had a mentor [to say] “Hey, I need you to do X, Y, and Z [or] you'll be better off doing this.” I’m grateful for the case managers who actually cared about my wellbeing, my son’s well-being. It was just a really good feeling and I'm just so happy. *24 years old, Some college, First HI 21 years old, Postpartum*
**Theme #2.2:** *Managing the pregnancy*	
Provide universal referral and care coordination for health care services, including prenatal care, to all pregnant people seeking homelessness and housing assistance.	It was hard to get prenatal care . . . I didn’t have an ID card, so I couldn’t get insurance . . . I did not go to the doctor. I tried to steal prenatal vitamins and I got caught . . . I didn't have food to feed myself, let alone the baby. *25 years old, Vocational, First HI 20 years old, Postpartum*
	I feel like they’re not worried about the person . . . they’re worried about getting you housed . . . [but] they don’t care where is that, how much it costs [you] . . . they’re not caring about is she going to be successful, is she going to have a case manager, making sure that she’s connected with her mental health person, is she going to have the food, is she going to be stable, is she going to have the support to be able to be a productive person in the community? *32 years old, Some college, First HI 30 years old, Postpartum*
	I was going into hospitals, screaming and crying saying “I’m pregnant, and I'm homeless, and I need help.” . . . because they weren’t doing anything in the shelter . . . I don’t feel safe in the streets. I don’t want to lose my baby. I’m not eating as I should every day and I don't have the proper resources for me and baby to stay healthy. If I didn’t go in there screaming and screaming and screaming about it, I probably would have lost the baby*. 34 years old, Associate’s degree, First HI 18 years old, Pregnant*
**Theme #2.3:** *Mental health challenges*	
Provide universal referral to mental health services to all pregnant people seeking homelessness and housing assistance.	I did stress a lot. I was in the hospital … [because] I was stressing too much . . . I just had to stop stressing because it’s not good for the baby . . . it’s so hard to do that when life is not great at all. *23 years old, Some college, First HI 18 years old, Postpartum*
	When [homelessness] has gotten the best of me and I have given up, I just shut down completely. I wasn’t saying anything . . . I wasn’t eating, I just was sleeping … I didn’t want to do nothing, [My son] noticed that . . . he’d show his little teeth or he’ll give me a kiss. . .trying to pull me out because he could sense that something was going on. *24 years old, High school, First HI 16 years old, Postpartum*
	Being homeless and pregnant comes with a lot of mental-health issues . . . you should automatically put mental health services with it . . . my mental health case manager [helped] me through a lot of things because I thought about suicide because I didn't think I was prepared enough for my daughter . . . She just gives me different ways that I can look at stuff, go about stuff, react to stuff. Now, I see a psychiatrist . . . I have postpartum depression really bad. *25 years old, Vocational training, First HI 20 years old, Postpartum*

#### Theme #2.1 Meeting basic needs

Meeting basic needs often felt insurmountable. Beyond housing, participants faced challenges including lack of transportation, food, childcare, clothing (to fit changing bodies), and regular bathing and hygiene. Not having an address to receive insurance and social service documents was a major barrier to accessing services. Maintaining employment was challenging for many, particularly given inadequate employment protections for part-time and casual workers. Food was a major source of stress for most participants, particularly in light of the nutritional needs of pregnancy and the strong desire to nourish themselves well. Shelters often lacked a refrigerator to store fresh food (produce, dairy, and meats), and when they did exist, items were often stolen from shared spaces. Participants believed that the lack of fresh, healthy, and/or preferred foods negatively impacted their pregnancy, contributing to anemia, excess or insufficient weight gain, and diabetic and hypertensive disorders of pregnancy. Some women did not know where to get food support, whereas others accessed shelters, soup kitchens, friends, or family, or asked passersby for help. Even among those eligible for the Supplemental Nutrition Assistance Program, cost was a barrier. Spending large amounts of time trying to meet basic needs was a strain on their mental and physical health and diminished their capacity to navigate the bureaucratic system requirements to access resources.

Participants suggested that adequate training for care coordinators/case managers can help to ensure pregnant people are connected to all available resources while being mindful of time-sensitive needs during pregnancy and postpartum (i.e., sonograms, diapers/wipes, safe sleep space for the infant). Technology (e.g., mobile app) may be able to facilitate identification of resources to meet needs (e.g., food, clothing, mental health, medical/prenatal care, and transportation).

#### Theme #2.2 Managing the pregnancy

Accessing prenatal care, while experiencing homelessness, was difficult. Some participants did not access health care services until well after the first trimester and/or utilized EDs rather than a regular primary care or obstetric provider. Many attributed pregnancy complications (e.g., miscarriage, preterm birth, fetal growth restriction, decreased fetal movement, and high blood pressure) to the stress of homelessness. Some women relied on the emergency room or labor and delivery triage to confirm fetal well-being and/or request housing support. Those receiving prenatal care had difficulties accessing ultrasounds and specialists because of lack of appointment availability, transportation, and childcare. Access to critical medications was interrupted for some owing to cost and insurance barriers (e.g., prior authorization, change of address, unclear eligibility). Others did not take medications as prescribed because of difficulty establishing routines in unstable environments. Health care access challenges extended to mental health, postpartum, and infant appointments.

Participants suggested that homelessness assistance offices maintain a list of health care and prenatal care providers who accept Medicaid and offer free/sliding scale care. They felt that an engaged care coordinator would check in regularly to see if the resident had initiated care and whether they needed any help overcoming barriers to accessing health care (transportation, childcare, etc.).

#### Theme #2.3 Mental health challenges

Homelessness during pregnancy is a stressful life event that may benefit from mental health support. Nearly all participants described the experience of homelessness and HI as persistent chronic stress that negatively impacted their physical and mental health. There was immense worry about the impact of the ongoing stress on the pregnancy, both related to stress as a contributor to medical complications (i.e., high blood pressure) and that the fetus was experiencing the stress along with the pregnant person. The mental health cost of homelessness persisted into the postpartum period with participants describing both shame that their child was exposed to homelessness, and also joy about their child who was a reason to persist. Many people who experience homelessness during pregnancy have complex, and often traumatic, life histories that may contribute to anxiety, depression, suicidality, substance use disorder, and acute stress responses.

Participants felt that anyone who was experiencing co-occurring pregnancy and homelessness would have mental health struggles. Several participants suggested that all people seeking homelessness assistance should be given referrals to accessible (free, sliding scale, or accepting of Medicaid) mental health providers. Participants emphasized the importance of a good match, a therapeutic relationship, and having someone who could help them think differently about a situation or provide advice (especially without family support).

**Policy Recommendation #3:** Ensure that DC residents have meaningful access to a living wage and affordable housing that is sustainable so that families can parent in safe and stable environments. See Table 4 for practice change recommendations and key quotes from participants.

**Table 4.** Policy Recommendation #3: Ensure That Residents Have Meaningful Access to Affordable, Sustained Housing so That Families Can Parent in Safe and Stable Environments

Practice change recommendations	Key quotes
**Theme #3.1:** *Pathways to long-term sustainable housing*	
Prioritize safe, stale, independent housing with minimal transitions for pregnant people and parents.	It was very difficult and stressful and scary because you never know where you’re going to be . . . I didn’t want to be out in the street with my own toddler . . . Where I’m living now you have to put in the work to get your own place and . . . in two years, if I don’t get stuff together, then I’m technically going to be back on the street and homeless. *19 years old, High school, First HI 18 years old, Postpartum*
	It was stressful every month. I would dread the thought of her coming over for that recertification process because . . . I didn’t know that they were going to give me extensions . . .. I just kept thinking, “Oh, we're about to be homeless again. We’re going to be stuck on the street. I’m not stable, what are we going to do when this term runs out?” *24 years old, High school, First HI 13 years old, Postpartum*
	I think it would have been most helpful [to get] a voucher because . . . I worked as much as I could and saved up as much as I could . . . I’m only 19, and I have two babies. It would be just nice if they have certain types of programs that will help you get affordable housing. *19 years old, High school, First HI 18 years old, Postpartum*
	I got accepted off of the waiting list for an apartment through Section 8, which is low-income housing for DC residents. The neighborhood is awful. I literally met someone on the way to turn in my money order that told me about someone that got killed outside of her window . . . why would I want to live somewhere like that? How are they giving us vouchers that are always in the dangerous places? . . . There’s no other places*. 24 years old, High school, First HI 13 years old, Postpartum*
**Theme #3.2:** *Support to achieve financial independence*	
Invest in programs that support pregnant people and parents such as paid parental leave, cash support, job training and placements, subsidized housing, and ongoing mental health resources.	If you don’t have enough financial backing . . . You going to end up back in the streets again after [a] year . . . [In] the rapid rehousing [program], you pay 40% of your income. Then the government helps with … 60% for a year. Then you go back to paying the normal market rate for the place you're living in. If . . . you lose your job or anything, you end up back in the street again [and] start the process all over. *25 years old, High school, First HI 23 years old, Postpartum*
	If I can’t have a consistent babysitter for the children, I can’t go to work. If I can’t go to work, then I can’t maintain my rent. If I’m staying with someone . . . first thing they want to do is have their hand out … That makes it harder to just maintain stability. And then, just not knowing how things are going to go in these programs that are supposed to be designed to assist us with getting that housing stability. *32 years old, Some college, First HI 4 years old, Postpartum*

#### Theme #3.1 Pathways to long-term sustainable housing

Pregnant people experiencing homelessness face immense challenges that often become more challenging and complex once they are parenting. Participants reported acute anxiety related to the possibility of moving between different shelters and housing units, and fear that they would lose access to long-term housing placements and other social supports. During pregnancy, the lack of access to family housing meant that many were unable to access a sense of security that would enable them to plan proactively for their own prenatal care and establish a safe home environment for an infant. Some residents described sleeping with their newborn in a car, couch-surfing, or staying in unsafe environments (e.g., substance use, secondhand smoke, vermin infestations) because they had not been able to obtain long-term housing while pregnant. The fear of housing instability gained new complexity as it was compounded with feelings of shame that they were not “good mothers” because they were exposing their infant or toddler to unstable and unsafe housing environments.

Participants strongly believe that families must have safe, stable, clean housing without fear of impending return to housing instability or homelessness. They shared a conviction that providing stability for families is a good way to support the physical and mental health of parents and children.

#### Theme #3.2 Support to achieve financial independence

The need for stable housing continues to feel acute for residents who are postpartum and parenting increasingly mobile and aware children. There was pervasive sense of ongoing vulnerability to becoming unhoused again and having to renavigate homelessness assistance—this time with a child. Postpartum participants described how navigating service access became more difficult once they had given birth because of difficulties in accessing parental leave, inability to obtain flexible employment to balance caregiving responsibilities, lack of employment protections related to caregiving, and an absence of affordable childcare. None of the participants reported receiving any parental leave assistance, and many described losing their part- or full-time employment because of missed work owing to childcare responsibilities (sick children, medical appointments, and daycare hours not aligned with work hours).

Participants recommended that there be more options to receive cash assistance so that they could prioritize their needs, and not live in a constant state of economic precarity. Participants hoped for more job training and help identifying job opportunities that would allow them to balance work with caregiving and not demand that they sacrifice one for the other.

## Discussion

This study was conducted by a collaborative of researchers, homelessness assistance service providers, and local government staff to understand how pregnant people experiencing homelessness in Washington, DC, navigate systems to meet their needs. Our findings confirm current research demonstrating the immense stress of concurrent homelessness and pregnancy and the need for compassionate and dignified services.^28^ Our results expand upon the global qualitative literature describing extreme vulnerability with action-oriented policy and practice solutions.^5,29^ A reproductive justice lens affirms that the right to have children must be paired with the right to live in a safe and stable environment conducive to a healthy pregnancy. Study participants clearly discerned how the chronic stress of housing instability, exacerbated by the anxiety of unclear timelines for permanent family housing, negatively impacted their pregnancy and increased medical and social risk.

The connections between homelessness and poor health outcomes that participants described, particularly the impact of extreme stress and inadequate nutrition, are well documented in the literature.^30–32^ Mental health disorders and homelessness are often co-occurring and bidirectional, exacerbating challenges with stable employment, substance use disorders, and the executive function required to secure housing.^6,33^ Mental health worsens with exposure to toxic stress, including HI and violence, which increases risk for prematurity, low birth weight, and hypertensive disorders of pregnancy.^32^ The fear of not knowing when they might obtain housing and anticipated poor treatment from service providers adversely affects mental health. People who receive compassionate support from housing and health advocates report improved mental health.^34^ Housing stability and social support are important predictors of overall health and well-being, which the participants deeply desire for themselves and their children.^35^

###  

#### Policy and practice change

The first area of policy and practice change emphasized by participants was the importance of early and accessible permanent family housing for pregnant people and elimination of barriers that impede access. Waiting until the third trimester for referrals to family housing means that pregnant people spend critical windows for fetal development and individual health in economic precarity while sheltering in unstable, and at times, unsafe housing.^36,37^ In 2022, DC implemented a pilot policy enabling all pregnant people to access low-barrier shelter, supportive services, and case management in their first and second trimesters, with additional levels of support for those with a high-risk pregnancy. Thanks to funding from the Health Resource Services Administration and other private donors, and informed by this study, Community of Hope has launched an innovative care coordinator program to ensure pregnant people experiencing homelessness achieve timely connection to health care and social support services. This program, called Housing Our Newborns, Empowering You (HONEY), enables residents to establish a supportive relationship with a designated care coordinator who can assist with the complex navigation required to access some services.

Existing research has found that pregnant people experiencing homelessness report stigmatizing and unsupportive care from health providers, whereas this study noted similar experiences with housing service staff.^38,39^ Achieving person-centered, compassionate, and respectful care is critical to ensure homelessness assistance services are meaningfully accessible for pregnant people.^37^ Respectful care is increasingly recognized as a critical component of high-quality perinatal health care, and our findings suggest that this extends to homelessness services.^40,41^ Health and social service providers must foster a culture of respect, ensuring that staff have adequate training, support, and accountability.^39^ People experiencing homelessness want to feel that service providers care about them as human beings, meeting the need for social support that helps navigate the complexities of living in poverty.^42^ Supporting people to obtain stable housing is a good long-term investment in physical and mental health. The HONEY program is also training homeless service provider staff on the perinatal period and educating prenatal care providers on the homelessness assistance system and trauma-informed care. This is a promising initiative that will be evaluated and, if effective, be considered for replication.

The second critical area for policy and practice change includes ensuring that pregnant people experiencing homelessness and HI are connected with health care (physical and mental) and can meet their basic needs for survival while receiving care with dignity. Policy and practice changes that improve access to housing support and provide care coordination, such as those described earlier, have the potential to mitigate the negative health and financial repercussions of HI, which can persist for generations.^40,41^

Building transparent, interdisciplinary, navigable systems can help mitigate the physiological, mental, and environmental stresses that accompany profound need.^43^ Integrated case management of health and homelessness services characterized by clear instructions, streamlined intake with assessment of basic needs, and warm hand-offs between agencies can help residents access services and feel cared for, reducing mental health strain and accelerating time to stability.^44^ Meeting survival needs with care is an antiracist practice that can help avoid reproducing and enforcing structural racism through bureaucratic processes—this is imperative in DC where the study sample of all Black women reflects the racial economic inequities persistent in the city.^45,46^ Navigating public assistance often co-occurs with cascading hardships—feeling like life is on hold, while awaiting housing and unmet needs for basic survival, and that stability is out of reach.^47^

Our third area of policy and practice change recommendations centers on ensuring social service systems are navigable and that there are pathways to housing stability and financial security for pregnant people and parents of young children. Programs that reduce housing transitions and progress to long-term housing security enable pregnant people to focus on their well-being beyond survival.^48^ During COVID-19, eviction moratoriums and eased requirements for cash assistance demonstrated that increased housing and financial stability improved physical and mental health.^39,42^ Navigating complex requirements for public assistance for housing and other services can lead to feelings of hopelessness, represent a high cognitive burden, and exacerbate mental health challenges.

Supporting people to obtain stable housing is a good investment in long-term physical and mental health. Housing First approaches recognize that permanent housing is a critical foundation to engage in health and other social services that would be well applied to pregnant people.^49^ Participants universally reported that they need housing first to care for themselves, a pregnancy, and their children. Innovative programs are occurring throughout the United States, which aim to address social and housing needs through Medicaid—promising programs that are limited by rising housing costs and lack of quality housing inventory.^50,51^ The use of housing vouchers has been demonstrated to improve parental stress by alleviating the cost burden of housing, improving the quality of housing accessed, and lessening reports of feeling unsafe in the neighborhood—all prevalent concerns among study participants.^52^ Economic justice, including affordable housing, living wages, and parental leave, is a critical intervention to achieving reproductive justice, thriving, and joy for all families.

### Limitations

Our study includes some limitations. Although the experience of concurrent homelessness and pregnancy is pervasive in the United States, particularly in metropolitan areas, the geographical specificity of our study may limit its transferability.^53^ All recruitment occurred through telephone or email, potentially excluding people without access to these technologies; however, recent research in the United States indicates that the vast majority of unhoused people have access to mobile phones, computers, and the internet.^54^ We recruited participants through homeless assistance providers, whose services included access to mobile phones and computers, or referral to internet access locations. The purposive sampling strategy may have introduced some selection bias, as participants were identified through homelessness services agencies and not randomly. Although we did not purposively sample for self-identified race or gender identity, all study participants identified as Black or African American women, which may limit its generalizability to other racial and gender-diverse groups.

### Implications for Health Equity

As part of the DC CASI initiative, this study applied a collaborative approach to engage clinical and housing service providers, researchers, and government agencies to rapidly solicit and translate insights from resident lived experiences into policy and practice change recommendations. Using resident perspectives to develop and inform strategic policy and practice recommendations exemplifies humility, power-sharing, and translational learning between the collaborative and the community, a vital aspect of health equity practice. Findings from this study informed the development of strategic recommendations, catalyzed a new model for multisector collaboration, and influenced a system-wide change in practice that expands access to robust housing supports for pregnant people.

Multisector collaboratives, such as DC CASI, can help identify areas of opportunity to improve systems, break traditional silos of service delivery and data collection, and develop recommendations for system enhancement that confront how structural inequities become reified. Legislative and regulatory policymakers can utilize the findings of multisector collaboratives that engage residents to develop and implement system-level solutions to improve the health of the communities.

## Supplementary Material

Supplementary Appendix S1

Supplementary Appendix S2

## Abbreviations Used

CASI: Calling All Sectors Initiative
COVID-19: Coronavirus Disease
DC: Washington DC
ED: Emergency Department
HI: housing insecurity
HONEY: Housing Our Newborns, Empowering You Program
OHE: DC Health Office of Health Equity
PhD: Doctor of Philosophy
